# Treatment of Strictures of the Urethra

**Published:** 1829-01

**Authors:** M. Dupuytren


### STRICTURES OF THE URETHRA.

*Treatment of Strictures of the Urethra.* By M. Dupuytren.
From a Correspondent.

The mode of curing strictures of the urethra by confining a *large* bougie at the anterior part of the obstruction, is still employed with undeviating success in the wards of M. Dupuytren and of M. Breschet at the Hôtel Dieu. The mere contact, accurately preserved for eight or ten days, often enables a catheter of the largest size to pass freely where the smallest bougie could not previously penetrate.

Although the effect would obviously be more speedy by the use of bougies with conoid extremities, capable of being wedged into the stricture, M. Dupuytren is indifferent to this advantage, from the certain fact that, sooner or later, the obstacle will be overcome without. Yet the tapered point would not only act on a larger surface at one time, but be less liable to displacement.

The bougies thus introduced are provided with four very narrow tapes or strings, whereby they are attached to a T bandage, surrounding the waist and under the scrotum. In their passage they are coiled, at equidistant points, round a ring which is placed over the body of the penis.

Where the introduction of a bougie is impeded by spasm anterior to the organic stricture, it is there fixed in the first instance. Thus, in a case of stricture in the membranous part of the urethra, wherein the urine flowed guttatim, the bougie was stopped at the fossa navicularis. It was, therefore, fixed at this part; and at the visit after the expiration of twenty-four hours, it was found to have overcome the obstacle.

The ordinary practice of introducing bougies, gradually increasing in size, on the principle of what M. Dupuytren (as we contend improperly,) calls the *mechanical,* in opposition to the other, which he designates by the term *vital* process, is also employed. In fact, although the confining of a bougie within the urethra may do well in hospital practice, yet circumstances frequently occur wherein this would not be admissible; nor can it be absolutely required, when any one may be taught to cure a stricture by the graduated bougie alone.

The bougies used at the Hôtel Dieu are principally of elastic gum, tapering from one extremity to the other, which is a very considerable improvement: thus a bougie which at one end shall have a sixth of an inch in diameter terminates at the other in a fine point, capable of being worked into the minutest passage. We can scarcely contemplate a case, unattended by spasm, where, with ordinary dexterity, the introduction of such a bougie into the stricture could be impeded; and, in nine cases out of ten of retention, this penetration alone will be sufficient to promote the flow of urine, on the withdrawing of the bougie.

It may be proper to make one remark respecting the modus operandi of the bougie, which M. Dupuytren professes not to understand, as his term *mechanical* implies. There is nothing mechanical or dilatable in the process, according to the ordinary acceptation of these terms. The friction or compression produced by the bougie excites the action of the vessels, which is followed by the absorption of the thickened urethral membranes. Friction and compression operate in the same manner in the resolution of tumors on the external surface of the body.

